# Epidemiological Data on Periodontitis in a Sample of the Bulgarian Population: A Retrospective Study

**DOI:** 10.7759/cureus.95172

**Published:** 2025-10-22

**Authors:** Teodora Bolyarova, Silviya Petkova

**Affiliations:** 1 Periodontology, Faculty of Dental Medicine, Medical University - Sofia, Sofia, BGR; 2 Periodontology, Biodent Dental Center, Sofia, BGR

**Keywords:** alveolar bone loss, periodontitis, radiography, smoking, systemic disease

## Abstract

Objectives

The aim of our study is to provide information on periodontitis among a convenient sample of the adult population in Bulgaria.

Methods

This retrospective study included patients who sought dental care at a private dental center. A total of 447 patients participated in the study (mean age: 49.30 years; SD ±15.76), of whom 193 (43.2%) were male and 254 (56.8%) were female. The methods used included a survey and radiographic examinations. Threshold values for alveolar bone loss were applied to determine the presence and severity of periodontitis.

Results

Of the individuals studied, 309 (69.1%) were diagnosed with periodontitis. Among them, 104 (23.3%) had mild periodontitis; 141 (31.5%) had moderate periodontitis; and 64 (14.3%) had severe periodontitis. A significant association was found between smoking and the presence of periodontitis (p < 0.001). The prevalence of periodontitis was significantly higher in patients with cardiovascular diseases, chronic obstructive pulmonary disease, rheumatoid arthritis, osteoporosis, and cancer, compared to healthy individuals (p < 0.001, p < 0.002, p = 0.014, p < 0.002, p < 0.001, and p < 0.001, respectively). A multivariate logistic regression analysis identified age and residence in rural areas as the strongest predictors of periodontitis (OR = 1.223; 95% CI: 1.169-1.280; p < 0.001 for age, and OR = 3.537; 95% CI: 1.361-9.190; p = 0.010 for rural areas).

Conclusions

Based on the obtained results, we recommend conducting a large-scale epidemiological study in the Bulgarian population and implementing measures to raise public awareness about periodontal diseases, their prevention, and their control.

## Introduction

Periodontitis is a chronic, multifactorial inflammatory disease associated with a dysbiotic plaque biofilm and characterized by progressive destruction of the tooth-supporting apparatus. The main features of periodontitis include loss of supporting tissues manifested as clinical attachment loss (CAL) and radiographically confirmed bone loss, presence of periodontal pockets, and bleeding on probing (BOP). The classification of periodontitis is based on stages defined by severity (according to attachment level, radiographic bone loss, and tooth loss), complexity, extent, and distribution [1].

With the increasing aging population, it is expected that periodontitis will affect more people in the coming decades, posing serious challenges to public health. Improving knowledge about periodontitis is essential to ensure better treatment, preventive strategies, and optimal oral health for patients. Analyses reveal a growing trend in observational studies assessing the link between periodontitis and systemic diseases, highlighting the negative impact of periodontitis on various systemic conditions, and the increasing interest in this connection [2].

According to the MeSH classification, an increasing number of publications are related to diseases and conditions, most frequently from the categories of nutritional and metabolic diseases, cardiovascular diseases (CVDs), female urogenital diseases and pregnancy complications, and musculoskeletal diseases. With the advent of personalized medicine, the medical community is striving to identify specific factors that may influence individual health. In this context, periodontitis is presented as a condition with significant systemic effects and is therefore increasingly included in holistic and personalized approaches to health.

Data obtained from studies on large population groups are essential to reveal prevalence and significant disease factors, explore mutual relationships and influences with other diseases, and develop prevention programs. The lack of epidemiological periodontal research in Bulgarian society limits the ability to develop treatment and prevention programs. Data on periodontal diseases at the national level, as well as the planning of health promotion activities, are needed.

One of the most objective examinations in dental medicine is radiographic examination, which provides information about the condition of hard dental tissues and surrounding structures. The two-dimensional radiographic image from panoramic, periapical, or bitewing radiographs - used for periodontal status assessment - allows determination of the presence of the lamina corticalis, width of the periodontal space, spongiosa density, root surface contour, and level of the interdental septum. Radiographically assessable bone loss, characteristic of periodontitis, allows for determining and comparing estimates of periodontitis prevalence from studies in different populations, as well as within the same populations at different times. Two-dimensional radiography provides information about bone loss in interproximal sites, where the disease usually starts and progresses most intensely.

The questionnaire method provides data on sociodemographic factors, individual habits, and concomitant systemic diseases. The analyses of the obtained data are used to assess the prevalence of periodontitis and related risk factors in the studied sample.

According to the National Statistical Institute, the population of Bulgaria in 2025 is approximately 6,500,000 people. Preliminary data obtained from a pilot study conducted in a private dental center involving a large group of individuals may provide valuable insights into the prevalence of periodontitis in a Bulgarian population. These findings could serve as a basis for designing a subsequent large-scale epidemiological study.

The aim of our retrospective study is to provide information on the prevalence of periodontitis among a sample of the Bulgarian population, combined with sociodemographic data, associated behavioral factors, and diseases, in order to assess the characteristics of periodontitis in the adult population of Bulgaria. The hypothesis investigated in this study is that the prevalence of periodontitis among the adult population in Bulgaria is influenced by various factors and is associated with systemic diseases, thereby forming a characteristic disease profile within the studied population.

## Materials and methods

**Figure 1:**
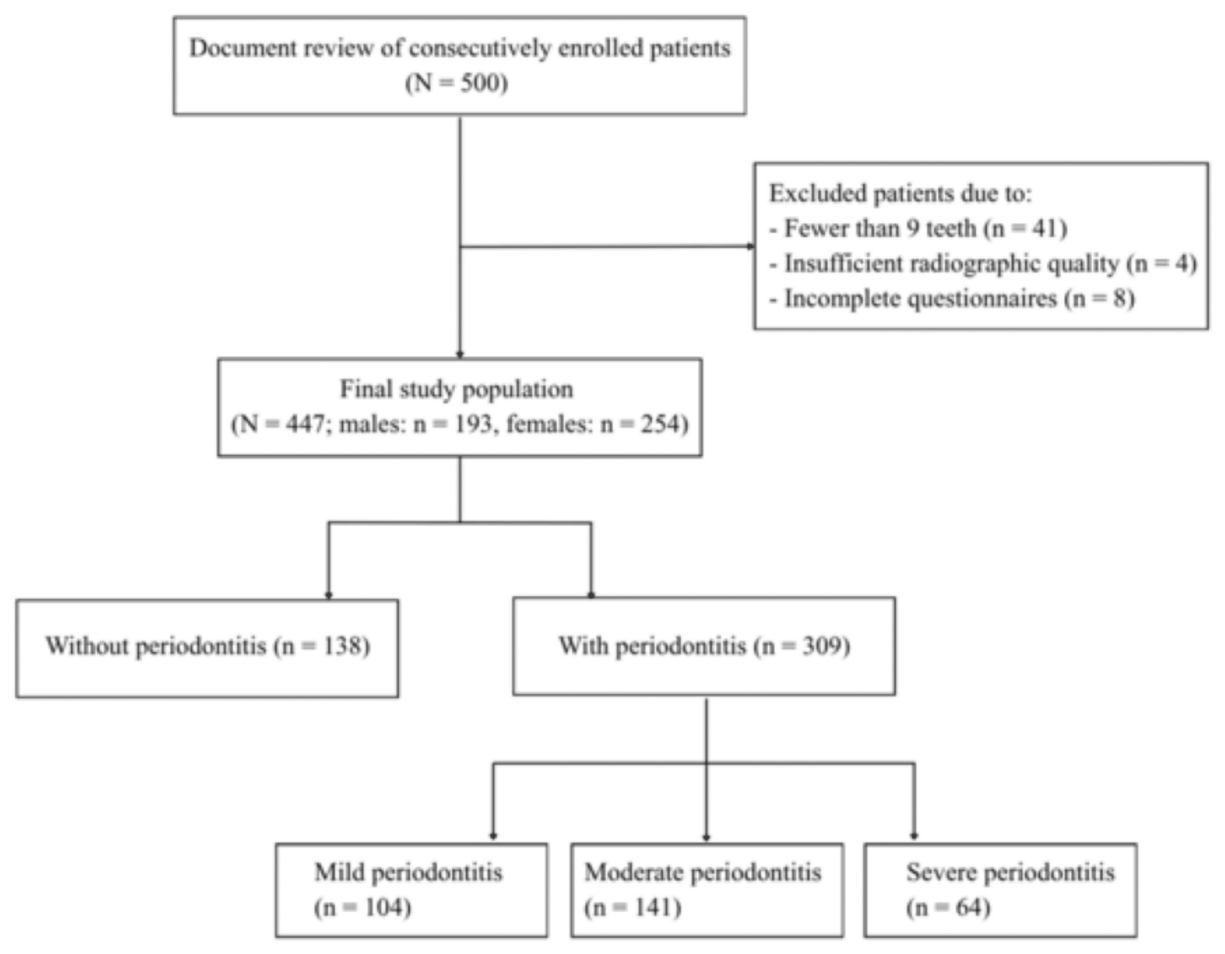
Flowchart of participant inclusion and exclusion

This retrospective descriptive study included patients who sought dental care for various dental and oral diseases between January 2021 and December 2022 at a private dental center in Sofia, Bulgaria. The study design, data collection, and reporting were conducted in accordance with the STROBE (Strengthening the Reporting of Observational Studies in Epidemiology) statement, ensuring transparency and methodological rigor in observational research. A total of 500 patients were consecutively recruited based on records documented at the time of their admission for examination and treatment. Inclusion criteria were age ≥18 years and having at least nine natural teeth. Patients with radiographs of inadequate quality or incomplete questionnaire data were excluded. Ultimately, 447 patients fulfilled the criteria and were enrolled in the study. The recruitment process, including the number and reasons for exclusions at each stage, is summarized in the flowchart (Figure 1).

The final study population consisted of 447 patients aged 18 to 89 years, with a mean age of 49.30 years (SD ±15.76). Of these, 193 (43.2%) were male and 254 (56.8%) were female (Table 1). Participants were recruited consecutively upon admission to the dental center, following a convenience sampling approach. No formal sample size calculation or statistical power analysis was performed prior to the study to determine the adequacy of the sample size, as the study aimed to generate initial observations to serve as a basis for future research.

**Table 1:** Demographic data of the study participants

Variable	Category	n	%
Gender	Female	254	56.80%
	Male	193	43.20%
Age group (years)	≤25	16	3.60%
	25-45	167	37.30%
	46-64	176	39.40%
	≥65	88	19.70%
Smoking status	Non-smokers	331	74.10%
	Former smokers	77	17.20%
	Current smokers	39	8.70%
Systemic diseases	Yes	396	88.60%
	No	51	11.40%
Place of residence	Capital	136	30.40%
	Large city (>100,000)	102	22.80%
	Small city (<100,000)	147	32.90%
	Rural area	62	13.90%
Total		447	100%

The research methods included conducting a survey with questions about demographic and social status, the presence of harmful habits, systemic diseases, as well as radiographic examinations. The questionnaire is provided in Appendix 1. Participants were queried regarding the presence of the following medical conditions: CVDs, chronic obstructive pulmonary disease (COPD), rheumatoid arthritis, osteoporosis, cancer, diabetes, kidney diseases, blood disorders, inflammatory bowel diseases, liver diseases, and infectious diseases. The exclusion criteria for this study comprised patients with radiographic images of insufficient quality, which precluded accurate assessment of bone loss, as well as patients who failed to provide adequate information in the questionnaires concerning harmful habits or systemic diseases. All patients received information and signed an informed consent form for the radiographic examination and the planned dental treatment.

Radiographic examinations performed for various indications, determined by the patient’s anamnesis and preliminary clinical dental examination, were used in the study following international radioprotection guidelines [3]. The imaging tests used were periapical radiographs and panoramic radiographs. The rationale for using radiographs was based on the established use of previous imaging for classifying periodontitis according to the AAP/EFP classification system for periodontal and peri-implant diseases and conditions [1]. The radiographic evaluation of alveolar bone loss was performed by two independent periodontology specialists with over five years of clinical experience. Prior to the assessment, the evaluators were calibrated to ensure consistency in interpretation and measurements. The radiographic assessments were carried out independently and blinded to the patients’ questionnaire responses and clinical status in order to minimize assessment bias. The imaging tests were thoroughly analyzed, considering the crestal bone both in terms of the presence of lamina dura and the distance to the cemento-enamel junction. Alveolar bone loss was calculated for each tooth as the distance in millimeters from the cemento-enamel junction to the alveolar bone edge minus 2 mm (normal alveolar bone distance) in the interproximal areas of all present teeth. Mean bone loss was calculated as the arithmetic mean of all values in the dentition. Threshold values of bone loss were used to determine the presence and severity of periodontitis. A case of periodontitis was defined as an individual with detectable radiographic bone loss measured on periapical radiographs and orthopantomograms of <15%, transformed into <1.5 mm at greater than or equal to two non-adjacent sites in the dentition, which cannot be attributed to causes unrelated to periodontitis. This definition and staging were based on the 2018 New Framework for applying the periodontal classification system to epidemiological data, according to which one of the criteria for epidemiologically defined periodontitis is bone loss <15% [4].

Mild periodontitis (Stage I) was defined as greater than or equal to two sites on non-adjacent teeth with radiographic bone loss <15% or <1.5 mm. Moderate periodontitis (Stage II) was defined as greater than or equal to two sites on non-adjacent teeth with radiographic bone loss of 15%-33% or ≥1.5 mm to <4 mm. Severe periodontitis (Stage III or IV) was defined as greater than or equal to two sites on non-adjacent teeth with radiographic bone loss >33% or ≥4 mm.

Data analysis

The study participants were consecutively recruited in the order of their admission for examination and treatment at the dental center. The participants were invited to complete a questionnaire providing information on demographic data, harmful habits, and general health status. Some of the questions were dichotomous variables, while others had multiple categorized response options. The answers were coded and entered into an Excel spreadsheet (Microsoft® Corp., Redmond, WA, USA). Bone loss values on the proximal surfaces of all present teeth were recorded from the taken and stored radiographs, and then the average value was calculated for each patient with periodontitis. Individuals without periodontitis were defined as those without bone loss.

Data were analyzed using IBM SPSS Statistics for Windows, Version 25 (Released 2017; IBM Corp., Armonk, NY, USA). Quantitative data were tested for normality, and descriptive statistics were presented according to the distribution. The following statistical methods were used for analysis: Spearman’s Rank Correlation, used to assess the relationship between two quantitative variables when the data are not normally distributed; Chi-square test (χ²), used to evaluate the association between two categorical (qualitative) variables, e.g., frequencies or percentages; Mann-Whitney U test, used to compare medians between two independent groups (for non-parametric data); Kruskal-Wallis test, used to compare medians among more than two groups (for non-parametric data); and Student’s t-test, used to compare means between two groups when the data are quantitative and normally distributed. The level of statistical significance was set at p < 0.05. We applied multivariate logistic regression analysis.

## Results

Prevalence of periodontitis and its association with various factors

In our study, periodontitis affected 309 individuals (69.1%), while 138 individuals (30.9%) had no periodontitis. The distribution of participants by periodontal status was as follows: 138 (30.9%) without periodontitis; 104 (23.3%) with mild periodontitis; 141 (31.5%) with moderate periodontitis; and 64 (14.3%) with severe periodontitis (Table 2). In 155 (50.2%) of the patients with periodontitis, bone loss was observed in more than 30% of the teeth, which is defined as generalized periodontitis.

**Table 2:** Prevalence and severity of periodontitis among the studied individuals

Presence and severity of periodontitis	n	%
Without periodontitis	138	30.9
Mild periodontitis	104	23.3
Moderate periodontitis	141	31.5
Severe periodontitis	64	14.3
Total	447	100

In the oldest age group (patients aged ≥65 years, total number = 88), the prevalence of severe periodontitis was highest (23.2%, n = 20).

A higher prevalence of periodontitis was found among men (71.2%, n = 137) compared to women (67.6%, n = 172), with no statistically significant difference between genders (p = 0.091, Student's t-test). According to our study, gender does not have a significant impact on bone loss prevalence. The mean bone loss from all periodontal sites in patients with periodontitis was 3.17 mm. Our study found a positive correlation between periodontitis prevalence, measured by bone loss, and increasing age (p < 0.001, Spearman’s rho = 0.682). Mean bone loss correlated positively with the number of missing teeth (p < 0.001, Spearman’s rho = 0.651). A statistically significant correlation was found between the number of missing teeth and increasing age (p < 0.001, Spearman’s rho = 0.648) (Table 3).

**Table 3:** Correlations between age, bone loss, and number of missing teeth

Variables	Statistical test	p-value	Coefficient/value
Age ↔ Bone loss (periodontitis)	Spearman's rank correlation	p < 0.001	ρ = 0.682
Bone loss ↔ Number of missing teeth	Spearman's rank correlation	p < 0.001	ρ = 0.651
Age ↔ Number of missing teeth	Spearman's rank correlation	p < 0.001	ρ = 0.648

Regarding place of residence, differences in periodontitis prevalence were observed (p < 0.001, Chi-square test, df = 9, Cramer’s V = 0.177). A large portion of individuals with periodontitis live in large cities (over 100,000 inhabitants), small towns (under 100,000 inhabitants), or villages - a total of 208 (67.4%) - and relatively fewer live in the capital city, 101 (32.6%).

Comparing mean bone loss values revealed a clear association between smoking and periodontitis presence (p < 0.001, Chi-square test, df = 1, Cramer’s V = 0.129). Among the patients who smoke (n = 39), severe periodontitis is more prevalent - in eight individuals (20.5%) - compared to the non-smoking patients (n = 331), of whom 37 (11.2%) have severe periodontitis. No association was found between alcohol consumption and the presence of bone loss.

Prevalence of periodontitis and severity of bone loss in relation to the presence of systemic diseases

Using questionnaire data, the association between periodontitis and systemic diseases was assessed. Among patients with CVDs, totaling 169 (37.8% of participants), 156 (92.3%) had periodontitis - a significantly higher proportion compared to the prevalence among individuals without CVD: 153 cases (55%) out of 278 participants (p < 0.001, Chi-square test, df = 1, Cramer's V = 0.393).

Of the patients with CVDs, 13 (7.7%) had no periodontitis; 43 (25.4%) had mild; 78 (46.2%) had moderate; and 35 (20.7%) had severe periodontitis (Table 4). Patients with CVD had significantly greater mean bone loss (2.96 mm) than healthy individuals (1.67 mm) (p < 0.001, Mann-Whitney U test) (Table 5).

**Table 4:** Prevalence and severity of periodontitis among patients with various systemic diseases Values are presented as n (%).

Systemic disease	Total with disease	Without periodontitis	Mild periodontitis	Moderate periodontitis	Severe periodontitis	Total with disease and periodontitis	p-value
Cardiovascular diseases	169 (37.8%)	13 (7.7%)	43 (25.4%)	78 (46.2%)	35 (20.7%)	156 (92.3%)	p < 0.001
Chronic obstructive pulmonary disease	19 (4.2%)	0	3 (15.8%)	13 (68.4%)	3 (15.8%)	19 (100%)	p = 0.002
Rheumatoid arthritis	24 (5.3%)	2 (8.3%)	6 (25%)	14 (58.4%)	2 (8.3%)	22 (91.7%)	p = 0.014
Osteoporosis	21 (4.7%)	0	3 (14.3%)	16 (76.2%)	2 (9.5%)	21 (100%)	p = 0.002
Cancer	22 (4.9%)	2 (9.1%)	4 (18.2%)	16 (72.7%)	0	20 (90.9%)	p < 0.001
Diabetes	23 (5.2%)	2 (8.7%)	5 (21.7%)	12 (52.2%)	4 (17.4%)	21 (91.3%)	p = 0.062

**Table 5:** Average bone loss in individuals with and without systemic diseases (mm)

Systemic disease	Mean bone loss with disease (mm)	Mean bone loss without disease (mm)	p-value
Cardiovascular diseases	2.96	1.67	p < 0.001
Chronic obstructive pulmonary disease	3.35	2.10	p = 0.002
Osteoporosis	3.12	2.11	p = 0.006
Diabetes	3.13	2.10	p = 0.011
Kidney diseases	2.80	2.11	p = 0.035
Liver diseases	3.36	2.13	p = 0.026

Among patients with COPD, totaling 19 individuals (4.2% of the examined population), periodontitis was present in all cases (100%), which is significantly higher compared to individuals without COPD - 290 individuals (67.8%) out of 428 (p < 0.002, Chi-square test, df = 1, Cramer's V = 0.141). In the group of patients with COPD, three (15.8%) had mild, 13 (68.4%) moderate, and three (15.8%) severe periodontitis. COPD patients had significantly higher mean bone loss (3.35 mm) than those without COPD (2.10 mm) (p = 0.002, Mann-Whitney U test).

Among patients with rheumatoid arthritis (n = 24; 5.3% of the examined subjects), periodontitis was present in 22 individuals (91.7%), which represents a significantly higher prevalence compared to individuals without rheumatoid arthritis - 287 individuals (67.8%) out of 423 were diagnosed with periodontitis (p = 0.014, Chi-square test, df = 1, Cramer’s V = 0.116). Among patients with rheumatoid arthritis, two (8.3%) had no periodontitis, six (25%) had mild periodontitis, 14 (58.4%) had moderate periodontitis, and two (8.3%) had severe periodontitis.

Among patients with osteoporosis (n = 21; 4.7% of the examined subjects), periodontitis was present in all individuals (100%), representing a significantly higher prevalence compared to individuals without osteoporosis, among whom 288 out of 426 (67.6%) had periodontitis (p = 0.002, Chi-square test, df = 1, Cramer’s V = 0.148). Among patients with osteoporosis, three (14.3%) had mild periodontitis, 16 (76.2%) had moderate periodontitis, and two (9.5%) had severe periodontitis. Osteoporosis patients had significantly greater mean bone loss (3.12 mm) than those without osteoporosis (2.11 mm) (p = 0.006, Mann-Whitney U test).

Among patients with cancer (n = 22; 4.9% of the examined subjects), periodontitis was present in 20 individuals (90.9%), which is significantly higher compared to individuals without cancer, among whom 289 out of 425 (68.0%) had periodontitis (p < 0.001, Chi-square test, df = 1, Cramer’s V = 0.107). Among patients with cancer, two (9.1%) had no periodontitis, four (18.2%) had mild periodontitis, 16 (72.7%) had moderate periodontitis, and none had severe periodontitis.

Among diabetic patients, there was a tendency toward a higher prevalence of periodontitis - 21 cases (91.3%) out of a total of 23 patients - compared to its prevalence among healthy individuals - 288 cases (67.9%) with periodontitis out of 424 non-diabetic individuals (p = 0.062, Chi-square test, df = 1, Cramer’s V = 0.112) - although the difference was not statistically significant. The lack of a clear correlation is likely due to the relatively small number of diabetic patients (n = 23), representing 5.2% of the examined subjects, compared to non-diabetics (n = 424). Among these patients, two (8.7%) had no periodontitis, five (21.7%) had mild periodontitis, 12 (52.2%) had moderate periodontitis, and four (17.4%) had severe periodontitis. Diabetics had significantly greater mean bone loss (3.13 mm) than non-diabetics (2.10 mm) (p = 0.011, Mann-Whitney U test).

The patients with kidney diseases - a total of 30 individuals, representing 6.7% of the examined subjects - had significantly greater mean bone loss (2.80 mm) compared to those without kidney diseases (2.11 mm) (p = 0.035, Mann-Whitney U test).

The patients with liver diseases - a total of 12 individuals, representing 2.68% of the examined subjects - had significantly greater mean bone loss (3.36 mm) compared to those without liver diseases (2.13 mm) (p = 0.026, Mann-Whitney U test).

Prevalence of periodontitis and severity of bone loss in patients with dental implants

Among the 58 patients with dental implants, who represent 13% of the total sample, periodontitis was present in 93.1% of cases (54 individuals), which is significantly higher compared to the 255 cases (65.6%) with periodontitis out of 389 individuals without implants (p < 0.001, Chi-square test, df = 1, Cramer’s V = 0.200). Among patients with dental implants, four (6.9%) had no periodontitis, 15 (25.9%) had mild periodontitis, 29 (50%) had moderate periodontitis, and 10 (17.2%) had severe periodontitis. Patients with dental implants had significantly greater mean bone loss (3.03 mm) compared to those without dental implants (2.03 mm) (p = 0.011, Kruskal-Wallis test).

A multivariate logistic regression analysis was conducted to identify independent factors associated with the presence of periodontitis in the studied population. After adjusting for potential confounding variables, age and residence in rural areas remained the strongest predictors of periodontitis. Age emerged as a significant independent risk factor, with each additional year associated with increased odds of having periodontitis (OR = 1.223; 95% CI: 1.169-1.280; p < 0.001). Similarly, living in rural areas was associated with a substantially higher risk compared to living in the capital or other major cities, with individuals residing in rural areas exhibiting more than a threefold increased risk (OR = 3.537; 95% CI: 1.361-9.190; p = 0.010). After further adjustment for sex in a separate model, the significance and strength of these main predictors remained virtually unchanged, indicating that these associations are not influenced by the participants’ sex.

This study does not provide information on gingivitis prevalence, which is a reversible disease, as the protocol did not include assessments of gingival health. Gingivitis is an inflammatory disease affecting only the free gingiva, without radiographic changes in the alveolar bone.

## Discussion

In relation to the role of dental medicine in global health, the need for action in three areas has been identified to improve oral health: (i) increasing knowledge in the field of epidemiology and health information systems; (ii) collecting, harmonizing, and strictly evaluating evidence for prevention, equity, and treatment; and (iii) developing optimal strategies for providing basic quality care to all those who need it without financial difficulties [5].

The need for research in these priority areas of dental medicine, along with the lack of epidemiological studies on periodontitis in Bulgarian society, provides grounds for conducting the present study. In it, we used retrospectively collected data on existing bone loss based on radiographs and questionnaire data. This study provides information on the prevalence and risk factors for periodontitis among a sample of the Bulgarian population.

When applying a threshold for radiographically detectable bone loss of 1.5 mm, we found that the prevalence of periodontitis was 69.1%, which is a high percentage compared to its prevalence in other European countries. In a study by König et al., CAL ≥4 mm was used as a criterion for measuring the prevalence of periodontitis in persons aged 35-44 and 65-74 years. According to this study, the prevalence of periodontitis varies across European countries, with the highest rates in Germany (>53.0% and >89.0% for the two age groups, respectively) and the lowest in Denmark (20% and 63.1%, respectively) and Spain (33.2% and 71.4%, respectively) [6]. Summarized data indicate that more than 50% of the European population suffers from some form of periodontitis, with over 10% having severe disease, and prevalence rising to 70%-85% among those aged 60-65 years. Periodontal health may worsen among the EU population, mainly due to a larger number of people retaining some of their teeth into old age and the increasing prevalence of diabetes [7].

When a threshold of radiographic bone loss >3 mm is applied, a study in a Norwegian population of 65-year-olds reported a periodontitis prevalence of 52.6%. However, when the threshold is lowered to >2 mm or >1 mm, the prevalence increases to 91.9% and 99.6%, respectively [8]. Our results show that if the bone loss threshold is raised to ≥1.5 mm, the prevalence of periodontitis decreases to 45.8%, and if the threshold is bone loss ≥4 mm, the prevalence is 14.3%.

Our results show that if the bone loss threshold for diagnosing periodontitis is raised to ≥1.5 mm, the prevalence decreases to 45.8%, corresponding to the proportion of participants with moderate and severe periodontitis - 31.5% (141 individuals) with bone loss between 1.5 mm and less than 4 mm, and 14.3% (64 individuals) with bone loss ≥4 mm, respectively. Furthermore, when the threshold is set at bone loss ≥4 mm, only 14.3% of individuals are classified as having periodontitis, reflecting the number of participants with severe periodontitis (64 individuals). These findings indicate the need to define a universal threshold for bone loss in epidemiological studies. However, using a higher threshold risks underestimating mild and moderate periodontitis.

Standardization of cases through radiographs was used in this pilot epidemiological study. It is not intended for clinical practice, where the diagnosis of periodontitis is primarily based on the clinical parameter of CAL. The imaging tests used are insufficient for diagnosis and are useful only for screening or recognition. We chose a lower threshold for detecting bone loss to avoid underestimating periodontitis.

Based on expert recommendations, and in order to more accurately assess the presence of periodontitis in a future large-scale epidemiological study, we propose the use of clinical measurements of periodontitis, such as CAL, probing pocket depth (PPD), and BOP [4]. These data will provide a more accurate assessment not only of the prevalence but also of the severity and activity of the disease, allowing for better planning of appropriate measures and resources for its prevention and control [9].

The results of our study show that the prevalence of periodontitis in the examined group is high - 69.1% of individuals were diagnosed with periodontitis (309 out of 447 participants). Of these, 14.3% (64 individuals) had severe periodontitis, while 54.8% (245 individuals) had non-severe forms, including moderate periodontitis (31.5%; 141 individuals) and mild periodontitis (23.3%; 104 individuals). These values are higher than those found in a nationally representative study conducted in the United States, which showed that 42.2% of dentate adults aged ≥30 years had some form of periodontitis, including 7.8% with severe periodontitis and 34.4% with non-severe periodontitis (moderate and mild), based on full-mouth periodontal examinations [10,11]. It can be assumed that infrequent dental visits, underdiagnosis of the disease, and the costs of periodontal treatment may be reasons for the high prevalence of periodontitis in the studied sample.

We found a higher prevalence of periodontitis in men (71.2%, 137 out of 193 individuals) compared to women (67.6%, 172 out of 254 individuals), although the difference was not statistically significant. Other studies indicate that men are significantly more likely to have periodontitis than women [10,11]. The mean bone loss was positively correlated with the number of missing teeth, which is expected, since both indicators reflect the extent of destruction and are used to determine disease severity per current classification.

In our study, the prevalence of periodontitis, determined based on the presence of bone loss, increases with age, which confirms the existing knowledge that the likelihood of having periodontitis gradually increases with advancing age [10]. According to a representative study from the United States, periodontitis is highly prevalent among the older age group (≥65 years), with nearly two-thirds of older adults with natural teeth being affected [10].

In our study, regarding place of residence, differences in the prevalence of periodontitis were also observed. A large proportion of individuals without periodontitis live in the capital city, while relatively fewer are from smaller settlements - towns or villages. The higher number of individuals without periodontitis in the capital is likely due to the proximity to dental clinics and the opportunity to receive preventive periodontal care. A similar association has been identified in the United States, where the distance to a dental specialist has also been recognized as a factor influencing the prevalence of periodontitis [10].

The results of the multivariate regression analysis indicate that the prevalence of periodontitis among the adult population sample in Bulgaria is strongly influenced by two factors - age and place of residence. Age is confirmed as an independent risk factor, likely as a result of the accumulation of factors associated with periodontitis. Individuals living in rural areas exhibit a significantly higher prevalence of the disease, which may reflect more limited access to dental care, lower awareness, or socioeconomic status. These findings underscore the importance of sociodemographic factors in the development of periodontitis.

The analysis showed statistically significant differences in the prevalence of periodontitis and mean bone loss among patients with certain systemic diseases and behavioral factors. The prevalence of periodontitis was also significantly higher in patients with CVD (92.3%) compared to those without the disease (55%); with COPD (100%) compared to those without it (67.8%); with rheumatoid arthritis (91.7%) compared to those without it (67.8%); with osteoporosis (100%) compared to those without it (67.6%); and with cancer (90.9%) compared to those without it (68.0%). A higher prevalence of severe periodontitis was observed among smokers (20.5%) compared to non-smokers (11.2%). According to other studies as well, periodontitis is significantly more likely to occur among current and former smokers compared to non-smokers [10,11].

The analysis demonstrated a statistically significant increase in mean bone loss among patients with certain systemic conditions compared to healthy individuals, as evidenced by higher mean bone loss in patients with CVDs (2.96 mm vs. 1.67 mm in individuals without the condition), COPD (3.35 mm vs. 2.10 mm), osteoporosis (3.12 mm vs. 2.11 mm), diabetes (3.13 mm vs. 2.10 mm), kidney diseases (2.80 mm vs. 2.11 mm), and liver diseases (3.36 mm vs. 2.13 mm). These findings are consistent with the large body of epidemiological, experimental, and interventional research that has shown that periodontitis may impact systemic health and is independently associated with most chronic non-communicable diseases. In recent years, however, significant scientific progress has been made - not only in the deeper understanding of the etiopathogenesis of periodontitis and the growing body of evidence supporting independent associations between periodontitis, diabetes, and CVDs, but also in relation to many other systemic conditions, including metabolic diseases and obesity, rheumatoid arthritis, certain types of cancer, respiratory diseases, and cognitive disorders, including Alzheimer’s disease [12].

It is now known that diabetes potentiates the severity of periodontitis and accelerates bone resorption. The risk and extent of alveolar bone loss are positively correlated with poor metabolic control [13]. Other studies have also found that individuals with CVDs showed a higher annual rate of bone loss compared to those without such conditions. Consequently, individuals with CVDs are at a higher risk of bone loss and periodontal diseases in general [14]. A review of the scientific literature from the past 25 years shows that systemically reduced bone mineral density in osteoporosis is associated with alveolar bone loss, and more recent data also suggest a correlation between CAL and other parameters of periodontitis. One of the possible mechanisms explaining the link between osteoporosis and periodontitis involves the presence of shared risk factors - primarily vitamin D deficiency and smoking [15]. Epidemiological and clinical studies have shown a positive correlation between periodontitis and COPD. The relative risk of COPD in patients with severe periodontitis is significantly higher compared to individuals without periodontal disease and those with mild to moderate periodontitis [16]. We found that periodontitis was more prevalent among patients with diabetes (91.3%) compared to non-diabetic individuals (67.9%), although the difference was not statistically significant. The lack of a clear correlation is attributed to the relatively small number of diabetic subjects compared to non-diabetic patients. Our study confirms the established association of periodontitis with several systemic diseases and conditions. A previous large-scale study, using multivariate logistic regression analysis, found a significant negative correlation between the scores of the eight essential health factors (Life's Essential 8, or LE8) and the prevalence of periodontitis. The eight essential health factors represent the latest indicator from the American Heart Association and are based on assessments of each component: nutrition, physical activity, nicotine exposure, sleep duration, body mass index, blood lipids, blood sugar, and blood pressure [17].

Our results, showing associations between prevalence of periodontitis, mean bone loss, and smoking and CVDs and diabetes - which are related to previous factors - support the established dependencies. Considering this, we deem it appropriate to include the assessment of the LE8 in future population-based periodontitis studies. The observed correlations between bone loss and common diseases, as well as smoking, justify the recommendation that patients with periodontitis should be evaluated for harmful habits and undergo additional tests, such as blood sugar measurement, lipid profile, and others, in relation to their overall health status. On the other hand, the lower prevalence of periodontitis among individuals without systemic diseases highlights the importance of prevention and control of periodontitis in relation to the manifestation of systemic diseases.

One of the purposes of epidemiological studies is to use their data to define steps for individual diagnosis. According to the Glossary of Periodontal Terms, periodontal diagnosis is the process (or the opinion resulting from the process) of determining the nature and cause of periodontal disease. The relevant information used in this process typically includes the application of medical and dental history, clinical and radiographic examination of the patient, as well as laboratory results [18]. Epidemiologically established data on risk factors and influences of periodontitis justify the incorporation of questions about general health and individual habits into the diagnostic process in clinical practice. In the present study, questionnaire data were collected and radiographic examinations performed for various indications, identifying risk factors for periodontitis. This can serve as the basis for developing a validated questionnaire for gathering data from patients with periodontitis and for considering therapeutic approaches. In studies on patients with periodontitis, we propose including questions about other systemic conditions and habits known to be associated with periodontitis, such as smoking, CVDs, rheumatoid arthritis, and others.

The presented data highlight the necessity of treating periodontitis in relation to preserving dentition, ensuring better quality of life, reducing economic costs for both the individual and society, and also maintaining and controlling systemic health.

According to a recent review study, the prevalence of periodontal diseases is decreasing, but aging populations and the increasing frequency of diabetes counterbalance this trend. An analysis of data in Germany shows a significant discrepancy between the diagnosis of periodontitis (approximately 25% of people with mandatory health insurance) and the provision of therapy (only about 2%). Therefore, despite a very high prevalence, there is a treatment gap. One possible reason is that periodontal inflammation does not cause pain, at least in its mild form. Another possible cause is a lack of trust in treatment outcomes. Continued information campaigns about the importance of oral hygiene and regular dental visits are expected to increase the percentage of patients seeking treatment in the coming years [19].

The results of our study indicate that patients with dental implants exhibited a significantly higher prevalence of periodontitis (93.1%) compared to individuals without implants, among whom 65.6% had periodontitis. This is likely due to the fact that, in most of our patients with implants, the preceding tooth loss was caused by periodontal disease. Our findings of greater mean bone loss in patients with implants (3.03 mm) compared to those without implants (2.03 mm) support the identification of periodontitis severity as a risk factor for tooth loss and the subsequent placement of dental implants. In the analysis of risk factors for peri-implantitis, contemporary dental literature highlights periodontitis and smoking as highly suggestive indicators. Additional suggestive risk factors include diabetes mellitus, hyperglycemia, lack of prophylaxis, and a history of chronic periodontal disease. These factors are also recognized as significant in the development and progression of periodontitis [20,21]. The control of risk factors, including effective management of periodontal disease, is essential for the long-term maintenance of dental implants.

Study limitations

This study is based on questionnaire responses and radiographic evaluation of the approximate levels of supporting alveolar bone in patients presenting to a dental practice for care related to various aspects of their dental status. One of the main limitations of the present study is that the diagnosis and severity of periodontitis are determined solely by radiographic evaluation, without performing a comprehensive clinical periodontal examination. Although radiographs provide valuable information about alveolar bone loss, they do not reflect the full clinical picture of periodontal disease, such as PPD, CAL, and BOP. This limitation may lead to underestimation or overestimation of the prevalence and severity of periodontitis.

Despite these limitations, the radiographic approach is justified by the retrospective nature of the study, and the availability of imaging data allows assessment of alveolar bone status in a relatively large patient sample. Future studies should combine radiographic data with detailed clinical periodontal examination to provide a more complete and accurate characterization of periodontal status. Limitations of this study include the relatively small number of patients for an epidemiological study and the absence of documented clinical periodontal examination in the examined patients. Another limitation is that the reasons for tooth loss were not determined; teeth were recorded as lost due to periodontitis when reduced bone levels were observed around the remaining teeth.

The reason for this is that the progression of periodontal disease leads to tissue destruction and subsequent tooth loss, and this sign is considered one of the indicators of disease severity according to the current classification of periodontitis. Furthermore, the percentage of patients with diabetes - a recognized risk factor for periodontitis - is lower in the current studied group compared to its prevalence in the general population, which may lead to underestimation of periodontitis prevalence and incorrect assessment of the mutual influence of the two diseases. Additionally, the retrospective nature of the study and the single-center approach, based entirely on data collected from a private dental center in Sofia, may introduce selection bias and may also limit the generalizability of the findings to the broader Bulgarian population.

It should also be noted that the established associations between periodontitis and smoking, as well as between periodontitis and systemic diseases, are based on a retrospective, cross-sectional study design and therefore do not demonstrate a causal relationship.

Strengths of the present study

To our knowledge, no epidemiological studies on periodontitis in the Bulgarian population have been found in the available literature; therefore, our results are unique and demonstrate public health relevance. The presented data from a study of individuals within the Bulgarian population reveal a high prevalence of periodontitis among patients visiting dental practices for various reasons. While the findings cannot be generalized to the entire Bulgarian population, they provide a valuable basis for planning larger-scale epidemiological studies.

It can be assumed that the number of people with teeth affected by periodontitis will increase, as people live longer and retain their teeth longer with age, and periodontitis is defined as a chronic disease that accumulates over a person’s lifetime. A future epidemiological study on periodontal diseases in Bulgaria should be considered a multicenter investigation, involving a statistically determined number of participants selected as a sample from the population, and should employ clinical periodontal examination in combination with paraclinical methods and questionnaire data. Our findings provide a valuable basis for planning larger-scale epidemiological studies and identify the risk factors and determinants of periodontitis in a Bulgarian population sample.

## Conclusions

The results from our pilot retrospective study demonstrate a high prevalence of periodontitis and a significant need for periodontal treatment among adults in Bulgaria. Since periodontitis is a public health issue due to its impact on quality of life, as well as its association with other common systemic diseases and conditions, it is of great importance to develop programs for its prevention and management, aiming for better control of systemic diseases. Based on the obtained results, a large-scale epidemiological study in the Bulgarian population could be considered, along with measures to raise public awareness about periodontal diseases. The next step would be to propose partial subsidization of periodontal treatment by the Bulgarian healthcare system.

## Appendices

Appendix 1

Name:

Personal ID No.:

Age:

Gender:

Phone:

Address:

Occupation:

E-mail:

Would you like to be reminded about your next appointment?

☐ No

☐ Yes - by: ☐ Reminder card ☐ E-mail ☐ Phone (morning/evening) ☐ SMS

How did you hear about us?

☐ From another patient/friend/relative __________________________

☐ From another dental practice ☐ Newspaper/Magazine ☐ Internet ☐ Other ____________

Name and phone number of a contact person in case of emergency: ________________

Medical History

Are you currently under medical supervision?

If YES, for what reason? ____________________________________________

Name and phone of your treating physician: ____________________________

Have you been hospitalized in the last 5 years?

☐ No ☐ Yes __________

Cardiovascular System

Have you had a heart attack?

☐ No ☐ Yes _______

Do you suffer or have you suffered from coronary artery disease/heart failure?

☐ Yes ☐ No

Do you suffer or have you suffered from mitral valve prolapse?

☐ Yes ☐ No

Do you have or have you had congenital heart disease?

☐ Yes ☐ No

Do you have damaged heart valves?

☐ Yes ☐ No

Do you suffer or have you suffered from heart murmurs?

☐ Yes ☐ No

Do you suffer or have you suffered from rheumatic heart disease?

☐ Yes ☐ No

Do you have a pacemaker?

☐ Yes ☐ No

Do you have problems with blood pressure?

☐ No ☐ Yes __________________

Central Nervous System

Do you suffer from epilepsy?

☐ Yes ☐ No

Do you suffer or have you suffered from emotional/psychological disorders?

☐ No ☐ Yes __________________

Respiratory System

Do you suffer or have you suffered from tuberculosis?

☐ Yes ☐ No

Do you suffer or have you suffered from chronic bronchitis, emphysema, or COPD?

☐ Yes ☐ No

Gastrointestinal System

Do you suffer from stomach ulcers?

☐ Yes ☐ No

Do you suffer or have you suffered from GERD (gastroesophageal reflux disease)?

☐ Yes ☐ No

Do you suffer or have you suffered from liver disease?

☐ No ☐ Yes __________________

Endocrine System

Do you have diabetes?

☐ No ☐ Yes, If Yes, please specify - controlled / uncontrolled? Type: __________________

Do you suffer from hypothyroidism or hyperthyroidism?

☐ No ☐ Yes __________________

Hematological System

Do you suffer or have you suffered from anemia?

☐ Yes ☐ No

Do you suffer from hemophilia?

☐ Yes ☐ No

Do you suffer from immunodeficiency conditions?

☐ Yes ☐ No

Allergies

Are you allergic to:

• Local anesthetics? ☐ Yes ☐ No

• Antibiotics? ☐ Yes ☐ No

• Latex? ☐ Yes ☐ No

• Other medications? ☐ No ☐ Yes __________

Do you suffer from seasonal allergies?

☐ Yes ☐ No

Do you suffer from asthma?

☐ Yes ☐ No

Are you allergic to foods or beverages?

☐ No ☐ Yes ____________

Are you allergic to insect stings (bees, wasps)?

☐ Yes ☐ No

Urinary System

Do you suffer or have you suffered from kidney disease?

☐ No ☐ Yes __________________

Are you on dialysis?

☐ Yes ☐ No

Musculoskeletal System

Do you suffer or have you suffered from:

→ Arthritis? ☐ Yes ☐ No

→ Inflammatory rheumatism? ☐ Yes ☐ No

→ Osteoporosis? ☐ Yes ☐ No

Do you have an artificial joint?

☐ Yes ☐ No

Have you taken or are you currently taking bisphosphonate medications (zoledronic acid, alendronate, ibandronate, risedronate, etc.)?

☐ Yes ☐ No

Others

Do you have or have you had a tumor or malignant disease?

☐ Yes ☐ No

Are you currently undergoing or have you undergone chemotherapy or radiotherapy?

☐ Yes ☐ No

Do you suffer from any illness not mentioned above?

☐ No ☐ Yes ___________

Do you drink alcohol?

☐ Yes ☐ No, If YES, how often and in what quantities? __________________________

Do you smoke?

☐ Yes ☐ No, If YES, how many cigarettes per day? __________________________

For Women

Are you pregnant or suspect you may be?

☐ Yes ☐ No

Are you breastfeeding?

☐ Yes ☐ No

Do you take oral contraceptives?

☐ Yes ☐ No

Are you undergoing hormone therapy?

☐ Yes ☐ No

Current Medications

Please list all medications you are currently taking, including dosage and frequency (including over-the-counter medicines):

Do you take any of the following medications?

If YES, please specify:

→ Antihypertensive drugs (for high blood pressure)? ☐ No ☐ Yes ______

→ Anticoagulants/antiplatelet agents (blood thinners)? ☐ No ☐ Yes ______

→ Tranquilizers? ☐ No ☐ Yes ______

→ Steroids? ☐ No ☐ Yes ______

→ Codeine or other narcotic medications? ☐ No ☐ Yes ______

By signing below, I certify that the information provided in this medical history form is true and complete. I agree to inform my dentist of any changes in my health status.

Date _____________________

Signature _____________________
